# BCG Induces Protection against *Mycobacterium tuberculosis* Infection in the Wistar Rat Model

**DOI:** 10.1371/journal.pone.0028082

**Published:** 2011-12-05

**Authors:** Amit Singhal, Vanessa Mathys, Mehdi Kiass, Colette Creusy, Baptiste Delaire, El Moukhtar Aliouat, Véronique Dartois, Gilla Kaplan, Pablo Bifani

**Affiliations:** 1 Novartis Institute for Tropical Diseases, Singapore, Singapore; 2 Communicable and Infectious Diseases, Scientific Institute of Public Health, Brussels, Belgium; 3 Groupe Hospitalier de l'Institut Catholique Lillois, Hôpital Saint Vincent, Université Catholique de Lille, Lille, France; 4 Department of Parasitology, Faculty of Biological and Pharmaceutical Sciences, University of Lille Nord de France, Lille, France; 5 Public Health Research Institute Center, University of Medicine and Dentistry of New Jersey, Newark, New Jersey, United States of America; 6 Singapore Immunology Network, A*Star, Singapore, Singapore; Fundació Institut d'Investigació en Ciències de la Salut Germans Trias i Pujol, Universitat Autònoma de Barcelona, CIBERES, Spain

## Abstract

Our understanding of the correlation of *Mycobacterium bovis* Bacille Calmette-Guerin (BCG)-mediated immune responses and protection against *Mycobacterium tuberculosis* (Mtb) infection is still limited. We have recently characterized a Wistar rat model of experimental tuberculosis (TB). In the present study, we evaluated the efficacy of BCG vaccination in this model. Upon Mtb challenge, BCG vaccinated rats controlled growth of the bacilli earlier than unvaccinated rats. Histopathology analysis of infected lungs demonstrated a reduced number of granulomatous lesions and lower parenchymal inflammation in vaccinated animals. Vaccine-mediated protection correlated with the rapid accumulation of antigen specific CD4^+^ and CD8^+^ T cells in the infected lungs. Immunohistochemistry further revealed higher number of CD8^+^ cells in the pulmonary granulomas of vaccinated animals. Evaluation of pulmonary immune responses in vaccinated and Mtb infected rats by real time PCR at day 15 post-challenge showed reduced expression of genes responsible for negative regulation of Th1 immune responses. Thus, early protection observed in BCG vaccinated rats correlated with a similarly timed shift of immunity towards the Th1 type response. Our data support the importance of (i) the Th1-Th2 balance in the control of mycobacterial infection and (ii) the value of the Wistar rats in understanding the biology of TB.

## Introduction

Tuberculosis (TB) remains a major challenge to public health world wide, with an estimated 2 million deaths annually and 2.2 billion people infected with latent *Mycobacterium tuberculosis* (Mtb) across the globe [1]. The only vaccine available in the clinic is live attenuated *M. bovis bacille Calmette-Geurin* (BCG), which was developed 90 years ago and is generally administered soon after birth [2]. BCG has been shown to be partially protective against active TB [3], [4], [5] and also against the more severe form of disease in young babies [6]. These effects of BCG are due to the induction of cell-mediated immune responses [7]. Analysis of mycobacteria-specific T cells secreting interferon-gamma (IFN-γ) is widely used as an indicator of vaccine efficacy. However, IFN-γ alone is not sufficient for protection. In fact we still do not fully understand how BCG modulates the immune response, resulting in its protective effect [2], [8].

Most of the information on vaccine efficacy was obtained from experiments conducted in the mouse and the guinea pig models of pulmonary TB [9]. Although the mouse model of TB is convenient due to the available immunologic reagents and easy bio-containment requirements [8], colony forming units (CFU) are reduced by only 1 log_10_ following BCG vaccination and the pathology of Mtb infection in the mouse lung is different from what is observed in humans [9]. BCG vaccinated guinea pigs, on the other hand, demonstrate a BCG vaccine-induced reduction in lung CFU ranging from 2–3 log_10_ [10]. Furthermore, guinea pig granulomas share similarities with humans and prevention of tissue damage can be easily assessed in this model [9], [10]. The disadvantage of the guinea pig is the limitation of available immunologic reagents and the exquisite susceptibility to Mtb infection. Indeed, in the guinea pig a single bacillus can cause fatal disease within several months [10], whereas the vast majority of humans can control Mtb infection [1]. We have recently characterized a model of experimental TB in the Wistar rat [11], [12]. Mtb infected Wistar rats develop well organized granulomas, a T helper type 1 (Th1) immune response, and control bacillary growth in lungs [12]. In some of the animals bacillary clearance is more extensive and subclinical infection is established [12].

Protection against Mtb infection involves the coordinated activation and maturation of many types of leukocytes. CD4^+^ and CD8^+^ T cells dominate this process of restricting exponential growth of bacilli [13]. This restriction is mainly mediated by the production of Th1 cytokines including IFN-γ and tumor necrosis factor alpha (TNF-α) by CD4^+^ T cells and cytotoxicity against infected macrophages mediated by CD8^+^ T cells [14], [15]. The balance between the Th1 cytokines (interleukin [IL]-12, IFN-γ) and inhibitory Th2 cytokines (IL-4, IL-10, IL-13) and Transforming Growth Factor beta (TGF-β) is of importance in determining the extent to which the host controls the infection [16]. There are many reports indicating a robust Th2 response during Mtb infection both in mice and humans [17], [18], [19], [20]. Apart from Th2 cytokines, IFN-γ-mediated immune responses against Mtb infection can also be dampened by type I IFNs and negative regulators of Janus kinase-signal transducer and activator of transcription (Jak-Stat) pathway [16], [21], [22].

In the present study we used experimental pulmonary infection of Wistar rats to determine the degree of BCG vaccine-induced protection against challenge with the Mtb strain W4, a member of the W-Beijing strain family [23]. The presence of antigen specific IFN-γ secreting CD4^+^ and CD8^+^ T cells in the lungs of Mtb challenged BCG vaccinated rats was analyzed and compared with unvaccinated animals. In addition, the expression of genes involved in Th1-Th2 polarization was evaluated to delineate the mechanism(s) that lead to improved restriction of bacillary growth in BCG vaccinated rat lungs.

## Results

### BCG vaccine induced protection against *M. tuberculosis* in Wistar rats

**Figure 1:**
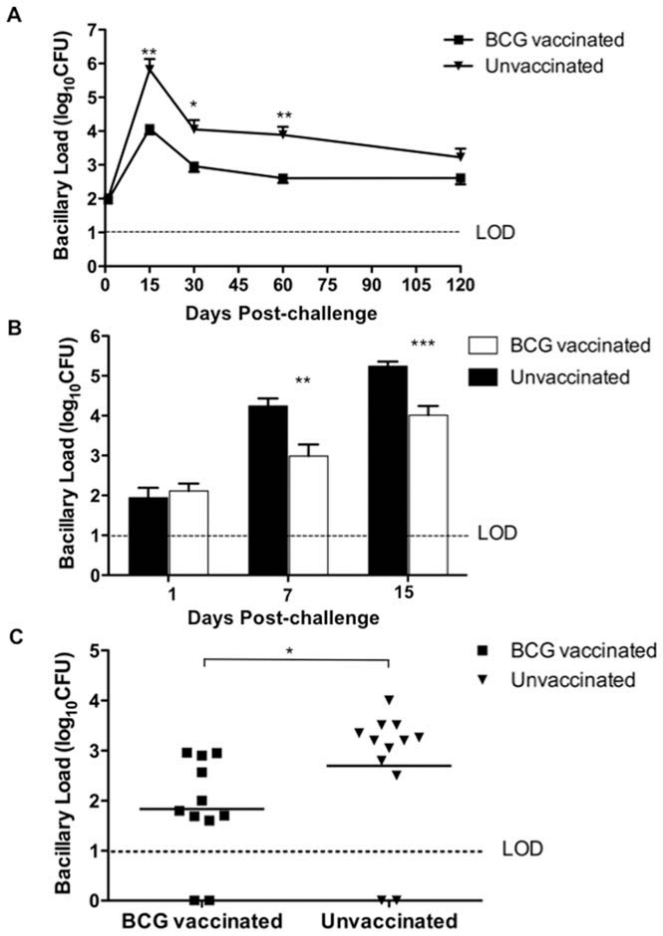
BCG vaccination of Wistar rats leads to protection early during infection. A) BCG vaccinated rats (N = 5) were challenged 6 weeks post vaccination with ∼100 CFU of Mtb W4 strain and the bacillary load in the lungs was evaluated till 120 days post-challenge and compared with that of age-matched unvaccinated rats. The difference in Mtb load between BCG vaccinated and unvaccinated group over time is significant (P<0.005, ANOVA). B) BCG vaccinated rats were challenged after 8 weeks of vaccination (N = 6) and infection was followed for 15 days. C) BCG vaccinated rats were challenged with Mtb after 6 weeks of vaccination and lung bacilli load at 120 days post-challenge was estimated and compared to that of unvaccinated rats. Values are means ± SDs (*, P<0.05; **, P<0.005; ***, P<0.0005; Mann-Whitney test).

We have recently shown that the Wistar rats can partially control low dose infection of Mtb and that the extent of control was a function of infecting dose and strain of Mtb used [12]. We examined whether BCG vaccination would enhance mycobacterial control in low dose Mtb infected rats. Animals were vaccinated with 10^6^ BCG subcutaneously and challenged with a low dose of Mtb W4 strain 6 weeks after vaccination. Age-matched rats received PBS instead of BCG and were used as unvaccinated controls. At the time of challenge, BCG vaccinated rats had no detectable BCG bacilli in the lungs (data not shown). Upon challenge, growth of Mtb W4 peaked at 15 days in the lungs of both vaccinated and unvaccinated rats (Figure 1A). However, a significantly reduced bacillary load was observed in the lungs of BCG vaccinated rats (approximately 10–50 fold less, P = 0.0079 by ANOVA) over the entire course of infection. (Figure 1A). We previously showed that under similar conditions the Mtb bacillary load in the spleen of Wistar rats is low, with almost no detectable CFU after day 60 post infection (p.i.) [12]. Consequently, spleen CFU was not estimated in this study.

Since BCG mediated control of Mtb was already seen at day 15 p.i. (Figure 1A), a second experiment was performed to study the protective efficacy of BCG within the first 15 days post-challenge. Rats were vaccinated and challenged after 8 weeks of vaccination. At 7 and 15 days post-challenge, the bacillary load in vaccinated rats was significantly lower compared to unvaccinated animals (Figure 1B, P<0.005 by Mann Whitney test). This indicated that the early growth of Mtb was inhibited in BCG vaccinated rats and that this differential in bacillary load was maintained throughout the experiment.

In our earlier study we observed complete bacillary clearance from the lung of some rats reaching CFU below the limit of detection (LOD) from 120 days p.i. [12]. We therefore examined whether BCG vaccination could induce complete clearance of Mtb W4 in lungs in a larger number of infected rat. Rats were vaccinated with BCG and infected by Mtb after 6 weeks post-vaccination. Animals were evaluated at 120 days p.i. for lung bacillary load. About a 1 log_10_ reduced bacillary load was observed in the lungs of vaccinated rats compared to unvaccinated animals (Figure 1C; P = 0.0175 by Mann Whitney test). The number of rats with undetectable CFU was 2/11 and 2/12 in BCG vaccinated and unvaccinated animals respectively. This suggested that BCG vaccination had a limited impact on W4 Mtb bacillary control and did not achieve complete control even in animals with low dose infection.

### Effect of BCG vaccination on lung immunopathology in infected rats

**Figure 2:**
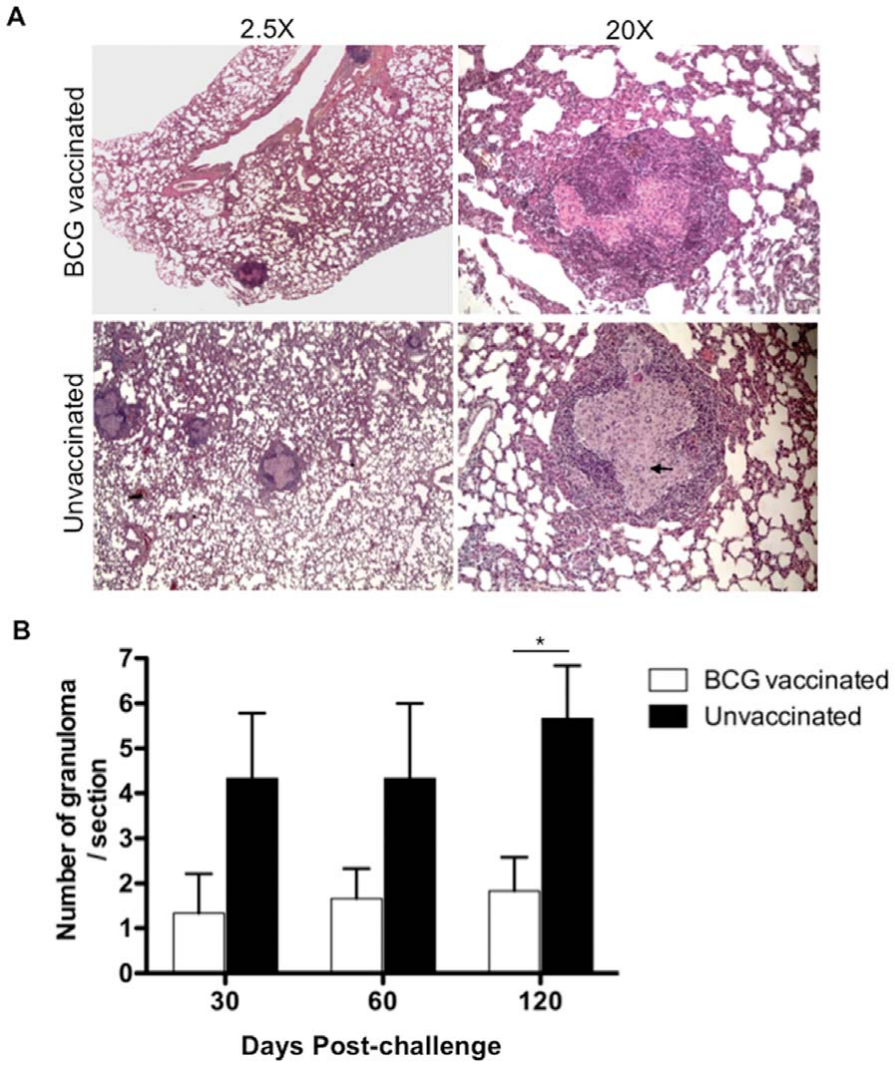
BCG vaccination of Wistar rat results in reduction in granulomatous inflammation in lung. A) Representative histopathologic micrographs of lungs of BCG vaccinated and unvaccinated rats challenged with Mtb W4 sacrificed at day 60 post-challenge. H&E, magnification 2.5× and 20×. BCG vaccinated rats have reduced number of granulomas and more normal lung parenchyma. B) Number of granulomas observed in 10 different lung sections per animal (N = 5 per group) at day 60 post-challenge. Values are means ± SDs (*, P<0.05).

**Figure 3:**
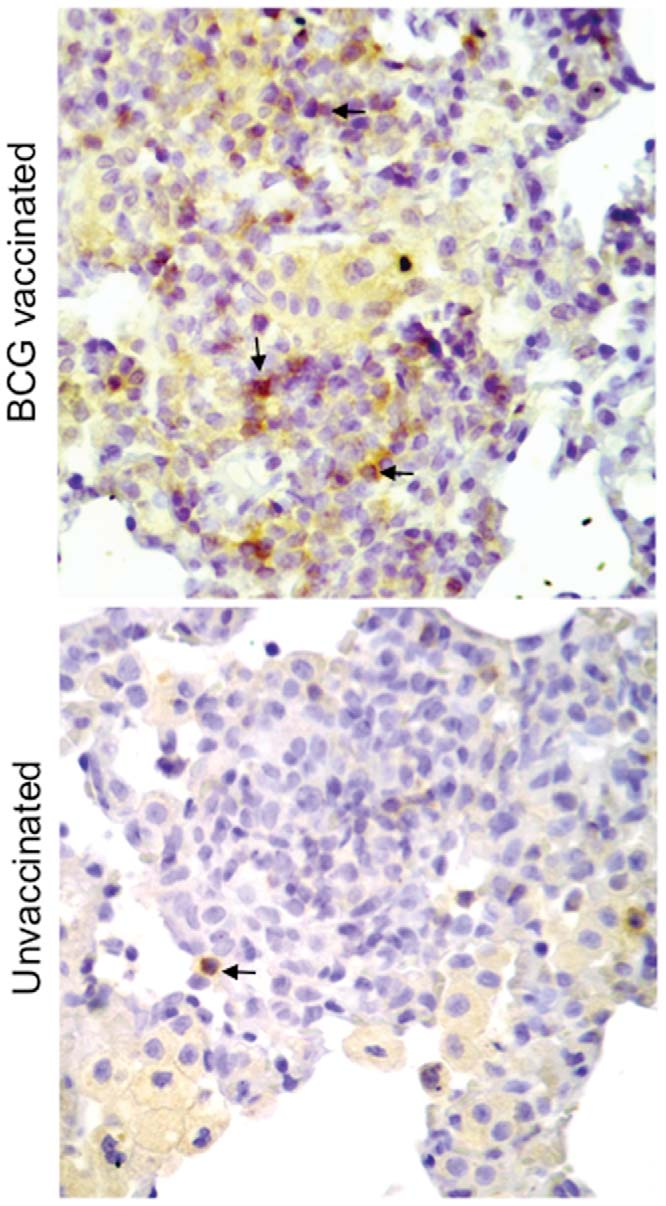
BCG vaccinated rats has more CD8^+^ cells in the lung granulomas. Representative micrographs of immunohistochemical staining of CD8**^+^ cells** in the lungs of BCG vaccinated and unvaccinated rats challenged with *M. tuberculosis* W4 and sacrificed at day 30 post-challenge, magnification 40×. Some of the positive cells have been indicated by an arrow.

Histologic examination of section of lungs from the different time points p.i. revealed that BCG vaccinated rats had significantly decreased pathology (Figure 2A). Further BCG vaccinated rats manifest fewer well organized granulomas in the lungs over the course of infection, compared to unvaccinated rats (Figure 2B). In both groups of rats at day 60 post-challenge the granulomas were well structured with a central area of epithelioid macrophages and a cuff of lymphocytes (Figure 2A). However, when the number of total cells and CD4^+^ and CD8^+^ T cells in the lungs was analyzed, a significantly greater number of total cells and CD8^+^ T cells was observed in BCG vaccinated animals at day 60 post-challenge compared with unvaccinated animals (Figure S1). Immunohistochemistry further revealed substantial higher number of CD8^+^ cells in the lung lesions of vaccinated rats at day 30 (Figure 3) and day 60 post-challenge (data not shown). Moreover, lung lesions from BCG vaccinated rats had small numbers of multi-nucleated giant cells as well (Figure 2A), a characteristic feature of the immune response in the Wistar rat TB model [11].

### Specific T cell responses in BCG-vaccinated and Mtb challenged rats

**Figure 4:**
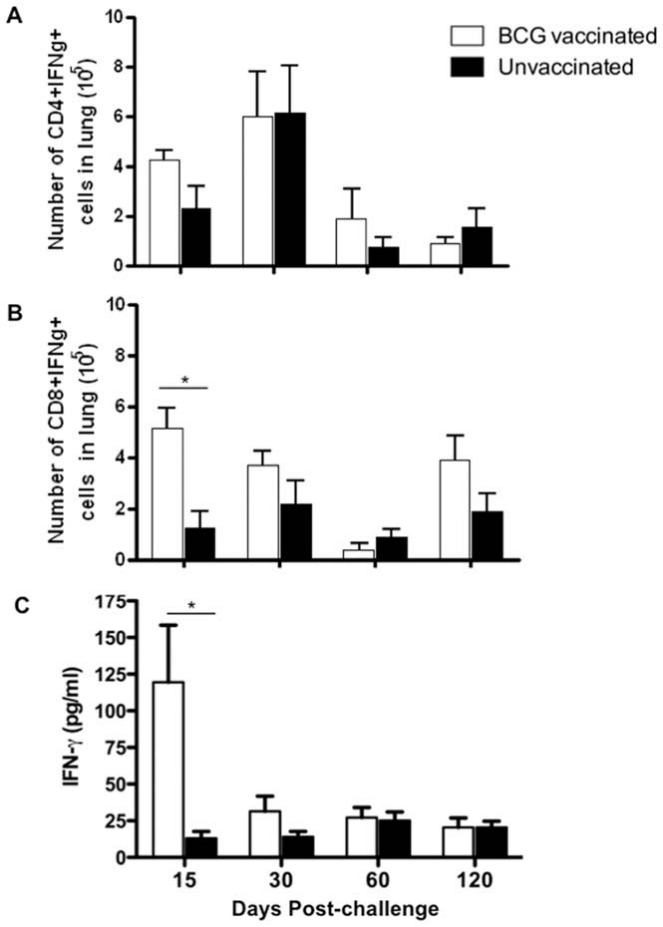
Specific T cell responses in BCG-vaccinated and Mtb challenged rats. Wistar rats were vaccinated with BCG. After 6 weeks vaccinated and age-matched unvaccinated rats were challenged with ∼100 CFU of Mtb W4. After 15, 30, 60 and 120 days post-challenge lung cells were isolated and exposed *ex vivo* to heat-killed Mtb and were analyzed for IFN-γ production as described in Materials and Methods. A) Total numbers of mycobacteria specific IFN-γ^+^CD4^+^ T cells in lung at the indicated days after Mtb challenge. B) Total numbers of mycobacteria specific IFN-γ^+^CD8^+^ T cells in lung at the indicated day after Mtb challenge. C) Amount of IFN-γ produced by lung cells upon ex vivo stimulation. Bars represent mean ± SD for cells from five individually analyzed rats (*, P<0.05, Student's t test).

The ability of BCG vaccination to elicit a specific T cell response prior to challenge was investigated. Lung cells from BCG vaccinated and unvaccinated rats were isolated after 6 weeks of vaccination, stimulated with heat-killed BCG and IFN-γ producing CD4^+^ and CD8^+^ T cells were enumerated. A high number of mycobacteria-specific CD4^+^ and CD8^+^ T cells producing IFN-γ was detected in BCG vaccinated rats (Figure S2). The number of CD8^+^IFN-γ^+^ cells was approximately 1.5 fold greater than that of CD4^+^IFN-γ^+^ cells. Next BCG vaccinated and unvaccinated rats were challenged with Mtb W4 and tested for the presence of IFN-γ producing T cells in the lungs. At 15 days post-challenge the number of IFN-γ producing CD4^+^ and CD8^+^ was higher in vaccinated rats compared with unvaccinated animal (Figure 4A and 4B). In vaccinated and unvaccinated animals specific CD4^+^ T cell responses in the lung peaked after one month of infection and then declined (Figure 4A). In contrast, CD8^+^ T cell responses in vaccinated rats remained somewhat elevated compared to unvaccinated animals over the course of infection except at day 60 post-challenge (Figure 4B).

To further investigate the T cell response, IFN-γ production was evaluated in culture supernatant of *ex vivo* stimulated lung mononuclear cells (Figure 4C). A significantly higher level of IFN-γ was produced by cells isolated from the lungs of BCG vaccinated rats at 15 days post-challenge compared to unvaccinated animals. Thereafter the IFN-γ levels from the cells isolated from BCG vaccinated rats declined. Overall early accumulation of IFN-γ producing mycobacteria-specific CD8^+^ T cells and IFN-γ production correlated with initial bacillary control rather than with bacterial load.

### Early post-challenge pulmonary immune responses in vaccinated rats

**Figure 5:**
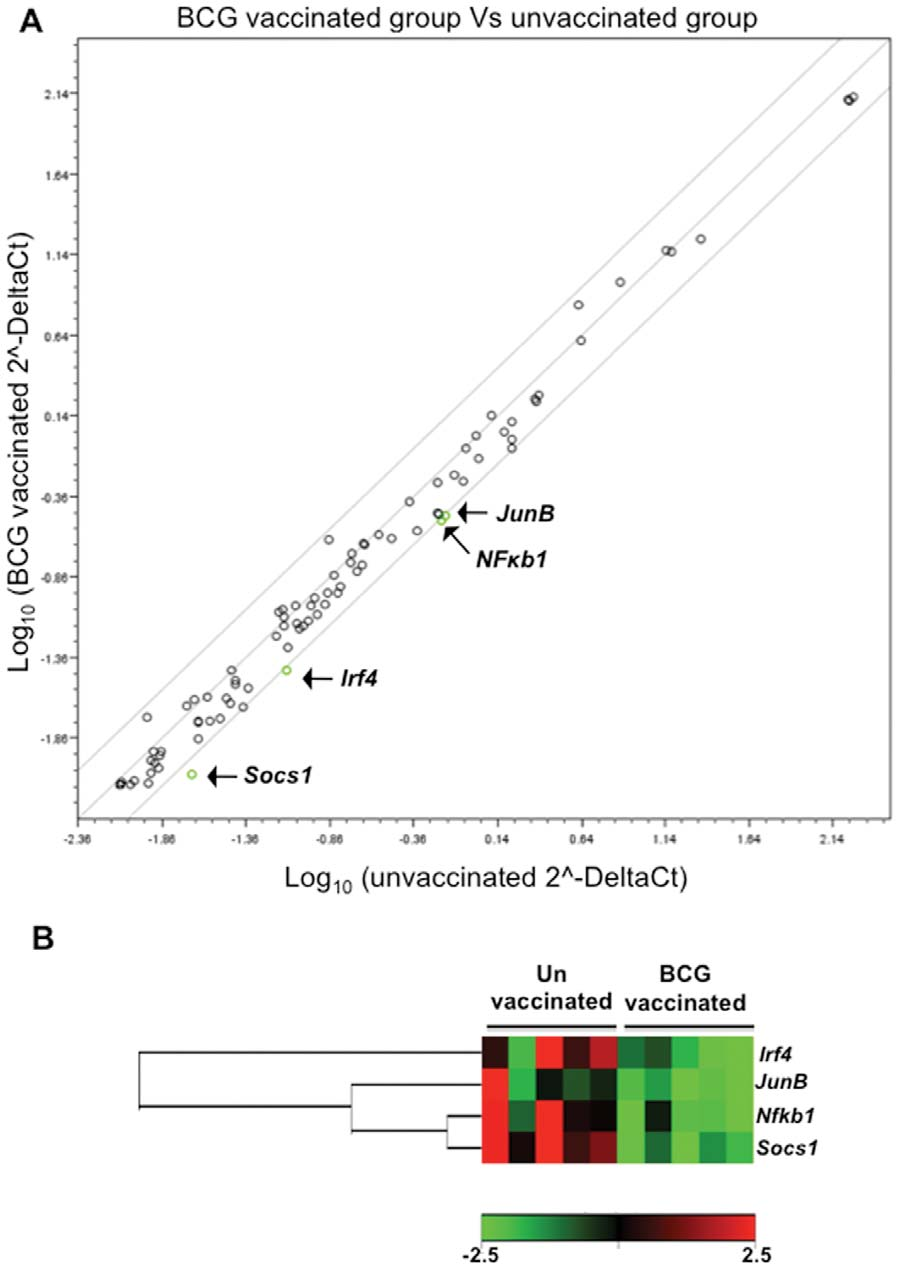
Quantitative PCR of RNA from lung cells demonstrates a decrease in negative regulators of Th1 immunity in Mtb challenged vaccinated rats. A) Total RNA from lung cells of Mtb challenged BCG vaccinated and unvaccinated rats (N = 5) was isolated at 15 days post-challenge and the relative expression levels for all 84 genes in the two groups are plotted against each other in the scatter plot. Genes encoding Socs1, Irf4, NF-κβ1 and JunB are down-regulated by at least two-fold in BCG vaccinated rats relative to unvaccinated rats (green circles). B) Cluster-diagram showing differential expression pattern of four of the above mentioned genes in individual rat belonging to the two treatment groups.

**Figure 6:**
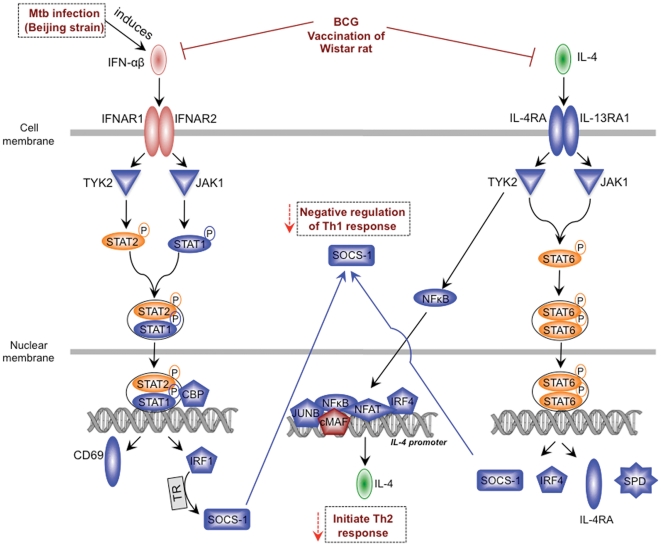
Hypothetical model of the regulation of IFN-αβ and IL-4 signaling in BCG vaccinated Mtb infected rats. Upon infection Beijing strains induce IFN-αβ [16]. BCG vaccination in Wistar rat inhibits the genes involved in IFN-αβ and IL-4 signaling and thus down regulates the negative regulators of Th1 immunity (indicated by red arrows). Downregulated genes observed in this study (Table S1) have been indicated in blue.

Since in our experiments BCG-induced protection peaked at 15 days post-challenge, RNA was isolated from lung cells of vaccinated and unvaccinated rats at this time point and real time PCR was carried out using a rat Th1-Th2-Th3 array as described in materials and methods. Out of 84 genes, significant differential expression (P<0.05) was observed for 20 genes between vaccinated and unvaccinated rats (Table S1). Out of these 20 genes, 17 were down-regulated and 3 were up-regulated in vaccinated compared with unvaccinated rats (Figure S3). Eleven of these 20 genes have been experimentally shown to have *NF-κB* binding sites in their promoters [24]. When an additional stringent criteria of at least two-fold regulation was applied to these 20 differentially expressed genes, only 4 i.e *Socs1* (Suppressor of cytokine signaling 1), *NF-κB1* (Nuclear factor of kappa light chain gene enhancer in B cells), *Irf4* (Interferon regulatory factor 4) and *JunB* (Jun-B oncogene), were selected (Figure 5A). All 4 genes were found to be downregulated in BCG vaccinated rats (Figure 5B). SOCS1 is a negative regulator of the Jak-Stat pathway that leads to the reduction of Th1 cytokines while the other 3 genes are transcription regulators that together are involved in the secretion of IL-4, a Th2 cytokine (Figure 6). In this experiment a two-fold upregulation of IFN-γ and Jak3 (Janus kinase 3) was also observed, though these values did not fall within the 95% confidence interval (p = 0.059 and p = 0.19 respectively, Table S1). Taken together the results of this study indicate the potential shift of the Th1-Th2 balance towards the Th1 response in association with early protective efficacy of BCG in Mtb W4 challenged Wistar rats.

## Discussion

The BCG vaccine-induced immune responses responsible for protection against Mtb challenge are very complex and not clearly understood. Most of the studies conducted in animal models and humans suggest the essential role of Th1 biased responses in vaccine-induced protection [25], [26], [27], [28]. However it has been shown, both in mice and humans, that level of CD4^+^ T cell IFN-γ production may not necessarily translate into immune correlates of protection against TB disease [8], [29]. In the present study we observed that the number of CD4^+^IFN-γ^+^ T cells was marginally higher, though not significant, in the lungs of vaccinated rats at day 15 post-challenge, but the kinetics of their accumulation in the lungs was similar to that of unvaccinated rats over the course of Mtb challenge. A greater number of activated CD4^+^ T cells has been observed in the lungs of BCG vaccinated guinea pigs within 15 days post-challenge [27], [30]. In contrast to CD4^+^ T cells, we observed faster and significantly higher accumulation of specific CD8^+^ T cells in the lung lesions of BCG-vaccinated rats. These CD8^+^ T cells were further observed to secrete more IFN-γ after 15 days of Mtb challenge. Rapid accumulation of specific CD8^+^ T cells and IFN-γ production have been observed in tissues of vaccinated and infected mice [8], [15] but the data on the recruitment of activated CD8^+^ T cells in BCG vaccinated guinea pigs after challenge is conflicting [27], [30], [31]. We also noted that the number of CD8^+^IFN-γ^+^ cells remained slightly higher in the lungs of vaccinated rats over the course of Mtb challenge. This indicates that in Wistar rats, the rapid CD8^+^ T cell accumulation and magnitude of specific CD8^+^ T cell responses and not specific CD4^+^ T cells, correlates with vaccine-induced sustained reductions in bacillary loads in the lungs of infected rats. A role for CD8^+^ T cells in anti-tuberculosis immunity has been demonstrated in a non-human primate model of tuberculosis [32], a model that closely resembles human TB [33]. Further it has been shown in human studies that Mtb specific CD8^+^ T cells possesses effector functions that could aid in the control of primary Mtb infection [34], [35].

Evaluation of BCG efficacy and other vaccine candidates of TB, is generally carried out by challenging animals with H37Rv strain. In this study we have used a strain belonging to the W-Beijing genotype of Mtb. This strain was selected due to the fact that Wistar rats clear H37Rv faster than the W4 W-Beijing strain [12]. Mtb strains of the W-Beijing genotype are among the most successful clinical isolates in humans, contributing significantly to the current TB epidemic [36]. This family of strains has often been associated with resistance to anti-TB drugs [37]. In the mouse model of pulmonary TB, strain-specific resistance to BCG-induced protective immunity has been shown to be uncommon [38]. However, in a rabbit model of TB meningitis (TBM), BCG vaccination was shown to confer poor protection against the Mtb W-Beijing strain HN878 [39]. In the present study BCG vaccination showed reasonably good protection in Wistar rats against the W-Beijing strain W4 i.e. a 1.5–2 log_10_ reduction in lung CFU. It is of interest that the HN878 and the W4 strain induce different responses in the pulmonary TB model of Wistar rats [12] as well as rabbits [39].

The hypervirulence of some W-Beijing strains appeared to be due to the failure of these strains to stimulate optimal Th1 type immunity [21], [40]. This has been found to be associated with induction of IFN-αβ and negative regulators of the Jak-Stat pathway in mice [16], [21]. In our earlier study we observed less IFN-γ production in rats infected with W-Beijing strains compared with those infected by H37Rv, which correlated with the differential control of the bacillary load in rats infected with these strains [12]. In the present study gene expression analysis of lung cells demonstrated significant down-regulation of 17 genes in vaccinated compared with unvaccinated rats; 14 of these 17 genes are potentially involved/related to the IFN-αβ and IL-4 signaling pathways (Figure S3). These 14 genes include *Socs1*, *NF-κB1*, *Irf4* and *JunB*, which displayed over a two-fold down-regulation in BCG-vaccinated rats. It has been shown earlier that induction of type I IFNs by W-Beijing strains lead to up-regulation of *Socs1* and other negative regulators of Jack-Stat pathway [16]. Further, SOCS1 has been shown to participate in the negative regulation of TLR signaling to dampen anti-mycobacterial activity [41], [42]. In TB patients, anti-TB drug treatment and killing of the tubercle bacilli results in a specific increase of Th1-type immune modulators (range of 3- to 14-fold) and decrease of mediators (including Socs1) that impair Th1-type immunity (range of 3- to 1,000-fold) following 15 or more days of treatment [43].

Along with *Socs1* expression of *Irf4* and *JunB*, transcription factors involved in the induction of IL-4 production, were found to be downregulated in the lungs of Mtb challenged BCG vaccinated rats [44], [45]. This indicates a possible inhibition of IL-4 signaling in vaccinated rats. Recently it has been shown that BCG vaccination reduce the expression of *IL-4* mRNA in lung and spleen of guinea pigs [46]. In our RT-PCR array experiment we did not observe significant reduction of *IL-4* expression in BCG vaccinated rats. Sufficient evidence has accumulated in the last decade indicating that IL-4 (and other Th2 derived cytokines) can undermine Th1 mediated immune response during TB and thus impair antimycobacterial immunity [47], [48], [49]. In humans with TB infection, increased expression of Th2-type cytokine mRNA has been noted in peripheral blood mononuclear cells (PBMCs) [47]. Upon treatment, disappearance of TB symptoms in patients has been correlated with a decrease in the relative amount of leukocyte IL-4 [19]. In mice BCG-CFP/CpG vaccine mediated protection has been shown to be associated with decreased IL-4, IL-5, IL-10, TNF-α and TGF-β concentrations, and reduced pathology [48].

In summary, we have shown that BCG vaccinated Wistar rats efficiently control early bacillary growth and pathology related to Mtb infection. The enhanced early protection could be due to a combined effect of increased IFN-γ producing CD8^+^ T cells and macrophage activation accompanied by down regulation of negative regulators of Th1 immune response. This inhibition of mediators of anti-Th1 immunity may leads to polarization of the immune response towards the Th1 type. Based on the results shown here and literature review, we propose a model of a mechanism of action of BCG vaccination mediated protection (Figure 6), wherein BCG vaccination leads to down regulation of the genes involved in IFN-αβ and IL-4 signaling pathways in the lungs of the W-Beijing Mtb strain challenged Wistar rats. This results in a shift of Th1-Th2 balance towards Th1 immunity and enhanced protection against Mtb infection. Further, the study also highlights the value of Wistar rats as an alternative model for testing vaccine efficacy against Mtb infection. In the Wistar rats we found that BCG efficacy is greater than mice and almost equivalent to guinea pig model. Further Wistar rat are used as the primary species for ADME (Absorption, Distribution, Metabolism and Excretion) and toxicology studies in early drug development. In our parallel ongoing drug studies we have observed that the relationship between *in vivo* efficacy and area under the curve/minimal inhibitory concentration (AUC/MIC) follows the same trend in the rat as for other animal models and in patients.

## Materials and Methods

### Ethics statement

All animals were maintained in accordance with protocols approved by the institutional animal ethical committee of ISP (Comité d'éthique concerté du CERVA, IPB at ISP). The authorization was approved by SPF Santé publique/FOD Volksgezondheid DG 4/Div 4 : Bien-être animal et CITES - Dierenwelzijn en CITES (No LA2230389).

### Bacterial strains

A well characterized clinical isolate of *M. tuberculosis* (Mtb), W/Beijing strain W4 (TB Center, Public Health Research Institute, UMDNJ, Newark, NJ, USA) [23] was used in the experiments. The strain was cultured in stationary Sauton medium in 500 ml cellular culture flasks to mid-log phase, washed, pelleted, re-suspended in phosphate-buffered saline (PBS), vortexed with 2 mm glass beads and prepared for infection as described [12]. For vaccination, *M. bovis* BCG strain Pasteur was cultured in Dubos broth base (Difco) supplemented with 10% Dubos medium albumin at 37°C. Mid-logarithmic culture was washed with PBS, aliquoted and stored at −80°C for use.

### Rats

Specific pathogen free (SPF) 6 week old outbred female Wistar Rats (Crl:WI) were purchased from Charles River, France. The animals were maintained in cages under biosafety-level-3 conditions at the Institute for Scientific Public Health (ISP), Brussels, Belgium. Throughout the experiments animals were fed sterile irradiated food and sterile water *ad libitum*.

### Vaccination, challenge of rats and enumeration of mycobacteria in lungs

Rats were vaccinated s/c with 10^6^ CFU of BCG. At 6–8 weeks after vaccination, animals were challenged with ∼100 CFU of *M. tuberculosis* W4 using an endoteacheal method as described [11], [12]. At indicated time points post-vaccination/infection rats were euthanized with sodium pentobarbital (55 mg/kg), lungs were aseptically excised and homogenized, followed by plating serial dilutions of homogenates on 7H11 agar plates and CFU enumeration after 3–4 weeks as described [12].

### Histopathology and morphometric evaluation of granulomas

Segments of the lungs (5 animal/time point) were fixed with 10% neutral formalin, embedded in paraffin and processed for histology. Sections (5 µm) were stained with (i) hematoxylin-eosin-safran (HES), and (ii) Ziehl-Neelsen stain for acid-fast bacilli (AFB). Histologic sections were used for morphologic analysis of the size and number of granulomas at different time points post infection.

### Immunohistochemistry of lung sections

5 µM sections (obtained as indicated above) on glass slides were deparaffinized in xylene and hydrated in water. The slides were treated with 0.3% H_2_O_2_ for 5 minutes to inactivate the endogenous peroxidase, followed by washing with PBS. The sections were incubated with the mouse anti-rat CD8 (AbD Serotec) followed by anti mouse-IgG HRP and developed using DAB (DAKO) using manufacturer's protocol. The sections were then counterstained with hematoxylin and examined by light microscopy.

### Lung cell digestion and flow cytometric analysis of lung cells

At the indicated time points following vaccination/challenge, rats were euthanized; lungs were perfused, aseptically removed from the pulmonary cavity, and single suspension was prepared as described [12]. The lung cells were incubated with Rat Fc block™ (BD Pharmingen) to block unspecific antibody binding. Subsequently, cells were stained with 0.2 µg of PEcy7-anti-CD4, FITC-anti-CD8α, or PE-anti-CD3 in fluorescence-activated cell sorting buffer (0.1% sodium azide, 1% bovine serum albumin) at 4°C. After two washes, the cells were fixed in 4% paraformaldehyde for 1 h and collected on a FACSCaliber (Beckon Dickinson). Analysis was performed by using CellQuest software (Pharmingen).

### *In-vitro* restimulation of cells and flow cytometric determination of cytokine expression

Cells (0.5×106) were cultured in a volume of 0.5 ml of RPMI medium 1640 supplemented with glutamine, Na-pyruvate, 2-mercaptoethanol, penicillin, streptomycin, and 10% heat-inactivated FCS and stimulated for 16 h with heat-killed W4 strain (MOI = 1). During the final 4 h of culture, 10 µg/ml brefeldin A were added. Cultured cells were washed and incubated Rat Fc blockTM (BD Pharmingen) to block unspecific antibody binding. Subsequently, cells were stained with FITC-anti-CD8α mAb and PECy7-anti-CD4 mAb (all BD Pharmingen, Franklin Lakes, NJ), and, after 20 min on ice, cells were washed with PBS and fixed and permeablized for 20 min on ice using Fix-perm buffer (Ctyofix/Cytoperm kit, BD Pharmingen). Cells were washed twice with 1× Perm buffer. This was followed by incubation with PE-conjugated anti-IFN-γ mAb in the presence of Rat Fc blockTM for 20 min on ice. Cells were washed and acquired on a FACSCaliber (Beckon Dickinson). Analysis was performed by using CellQuest software (Pharmingen).

### *In vitro* interferon gamma IFN-γ measurement

Lung cells (0.5×10^6^ cells) were incubated with heat-killed Mtb W4 (MOI = 1) for 72 hr at 37°C in 5% CO_2_-95% air. After incubation, supernatants were frozen at −80°C until processed for IFN-γ measurement using an ELISA kit according to the manufacturer's instruction (eBioscience).

### RNA isolation and evaluation of pulmonary responses by real-time PCR

To evaluate postchallenge immune responses, which may contribute to anti-TB protective immunity, lung cells from BCG-vaccinated and Mtb challenged rats (n = 5) was suspended in Trizol (Invitrogen) and total RNA was isolated from lung cell suspensions as per manufacturer instructions. Equal amounts of RNA from each sample were then reverse transcribed to cDNA by using a first-strand synthesis kit (Sabbiosciences, Frederick, MD) and measurement of changes in the expression of 84 cytokine genes representative of Th1, Th2 and Th3 cells were performed using rat Th1-Th2-Th3 RT^2^ Profiler™ PCR array according to the manufacturer's protocols (Sabbiosciences). The mRNA expression levels obtained for each gene was normalized to that of house keeping genes and ΔΔCt based fold-changes was calculated for each gene using web based PCR array data analysis software.

(http://pcrdataanalysis.sabiosciences.com/pcr/arrayanalysis.php)

### Statistical analysis

All values were expressed as means ± SD or means ± SE. Differences between the different groups in the protection experiments were assessed by one-way ANOVA of the log_10_ CFUs followed by Tukey's test and also by nonparametric Mann-Whitney *U* test (for comparison of two groups). The data obtained from immunological assays were analyzed using a Student's *t*-test. The statistical analysis of RT-PCR assay was carried out using online software at Sabbiosciences website. All statistical analysis was carried out using GraphPad PRISM software. A value of P<0.05 was considered significant.

## Supporting Information

Figure S1 **Influx of T cells in the lungs at 60 days post-challenge.** Wistar rats were vaccinated with BCG and after 6 weeks vaccinated and age-matched unvaccinated rats were challenged with ∼100 CFU of Mtb W4. At 60 days post-challenge number of total T cells, total CD3^+^CD4^+^ and total CD3^+^CD8^+^ cells was assessed in the lung. Bars show mean ± SM for cells from five individually analyzed rats. *, P<0.05; **, P<0.005.
(PDF)

Figure S2 **Specific T cell responses in BCG vaccinated rats.** Wistar rats were sub-cutaneously vaccinated by 10^6^ BCG. After 6 weeks rats were sacrificed and lung cells were isolated. Lung cells from BCG vaccinated and age matched unvaccinated controls were restimulated with heat killed BCG overnight and cells were analyzed for IFN-γ^+^ T cells as described in Materials and Methods. Graph shows total number of IFN-γ^+^ CD4^+^ and CD8^+^ cells in the lung. Bars give mean ± SEM for cells from five individually analyzed rat. In this experiment no BCG could be retrieved from the lung homogenate. *, P<0.05; **, P<0.005.
(PDF)

Figure S3 **Cluster diagram of 20 genes, fold difference of which was statistically significant (P<0.05) irrespective of their fold change (look Table S1), N = 5.**
(PDF)

Table S1 **Fold regulation in the gene expression in the lung of BCG vaccinated rats compared to unvaccinated rats.** Fold regulation and p value for each gene in the RT2 Profiler PCR Array Rat Th1-Th2-Th3 (Cat No. PARN-034A) has been presented. Postively and negatively regulated genes in BCG vaccinated rats compared to unvaccinated are shown in red and blue respectively. Genes for which p value was significant was taken into consideration.
(PDF)
