# Supplementary figures and images for: BCG Induces Protection against Mycobacterium tuberculosis Infection in the Wistar Rat Model
Source: PLoS One. 2011 Dec 5;6(12):e28082. doi: 10.1371/journal.pone.0028082 (PMC3230592; doi:10.1371/journal.pone.0028082)

Figure S1

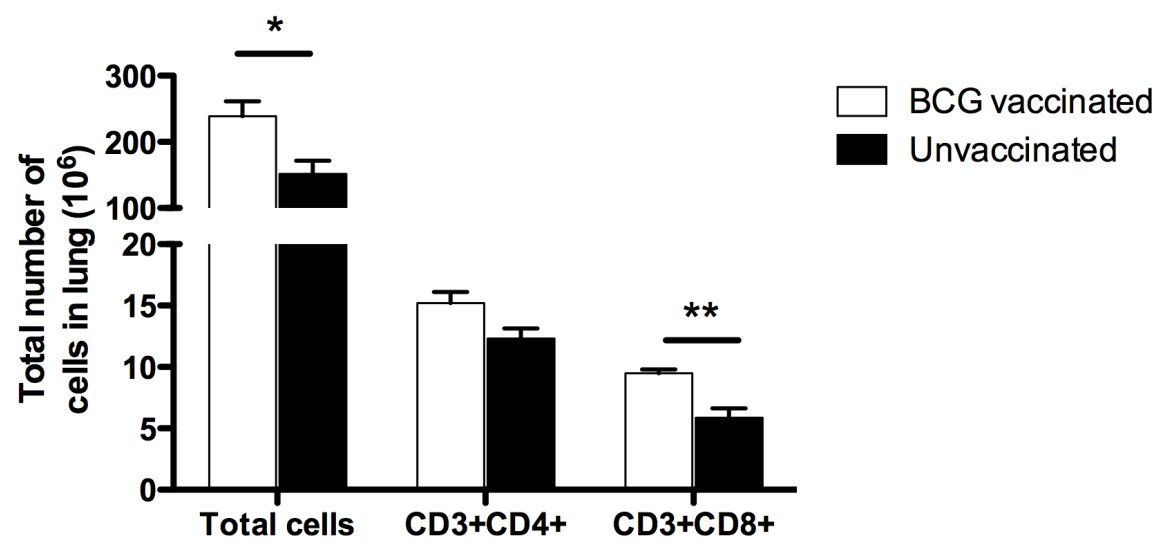

Supplement: Figure S1 — Influx of T cells in the lungs at 60 days post-challenge. Wistar rats were vaccinated with BCG and after 6 weeks vaccinated and age-matched unvaccinated rats were challenged with ∼100 CFU of Mtb W4. At 60 days post-challenge number of total T cells, total CD3+CD4+ and total CD3+CD8+ cells was assessed in the lung. Bars show mean ± SM for cells from five individually analyzed rats. *, P<0.05; **, P<0.005. (PDF) [file pone.0028082.s001.pdf]

Figure S2

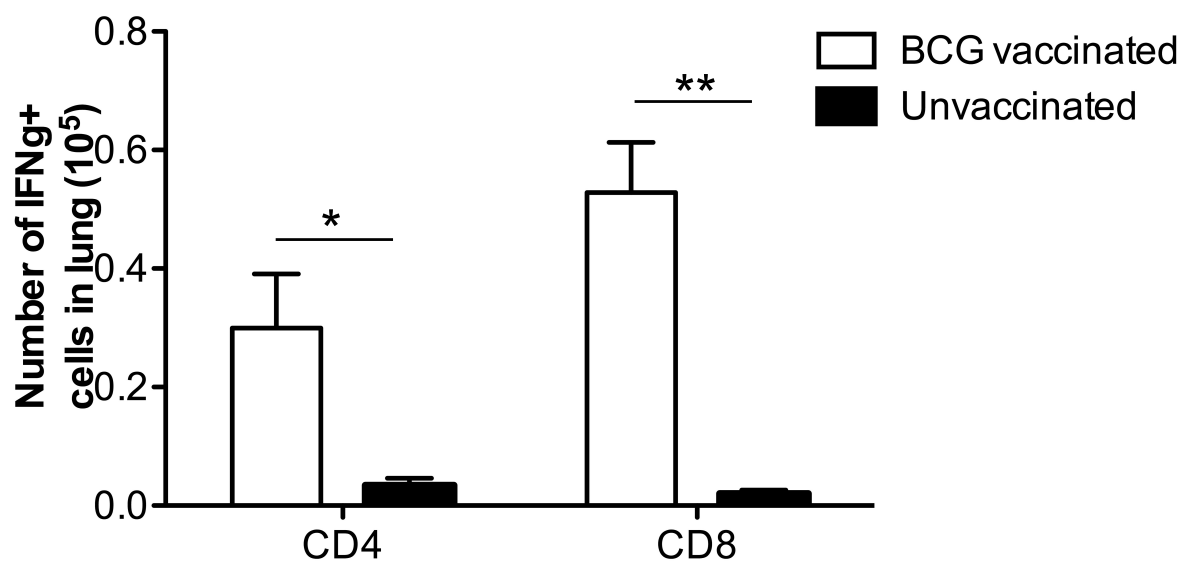

Supplement: Figure S2 — Specific T cell responses in BCG vaccinated rats. Wistar rats were sub-cutaneously vaccinated by 106 BCG. After 6 weeks rats were sacrificed and lung cells were isolated. Lung cells from BCG vaccinated and age matched unvaccinated controls were restimulated with heat killed BCG overnight and cells were analyzed for IFN-γ+ T cells as described in Materials and Methods. Graph shows total number of IFN-γ+ CD4+ and CD8+ cells in the lung. Bars give mean ± SEM for cells from five individually analyzed rat. In this experiment no BCG could be retrieved from the lung homogenate. *, P<0.05; **, P<0.005. (PDF) [file pone.0028082.s002.pdf]

Figure S3

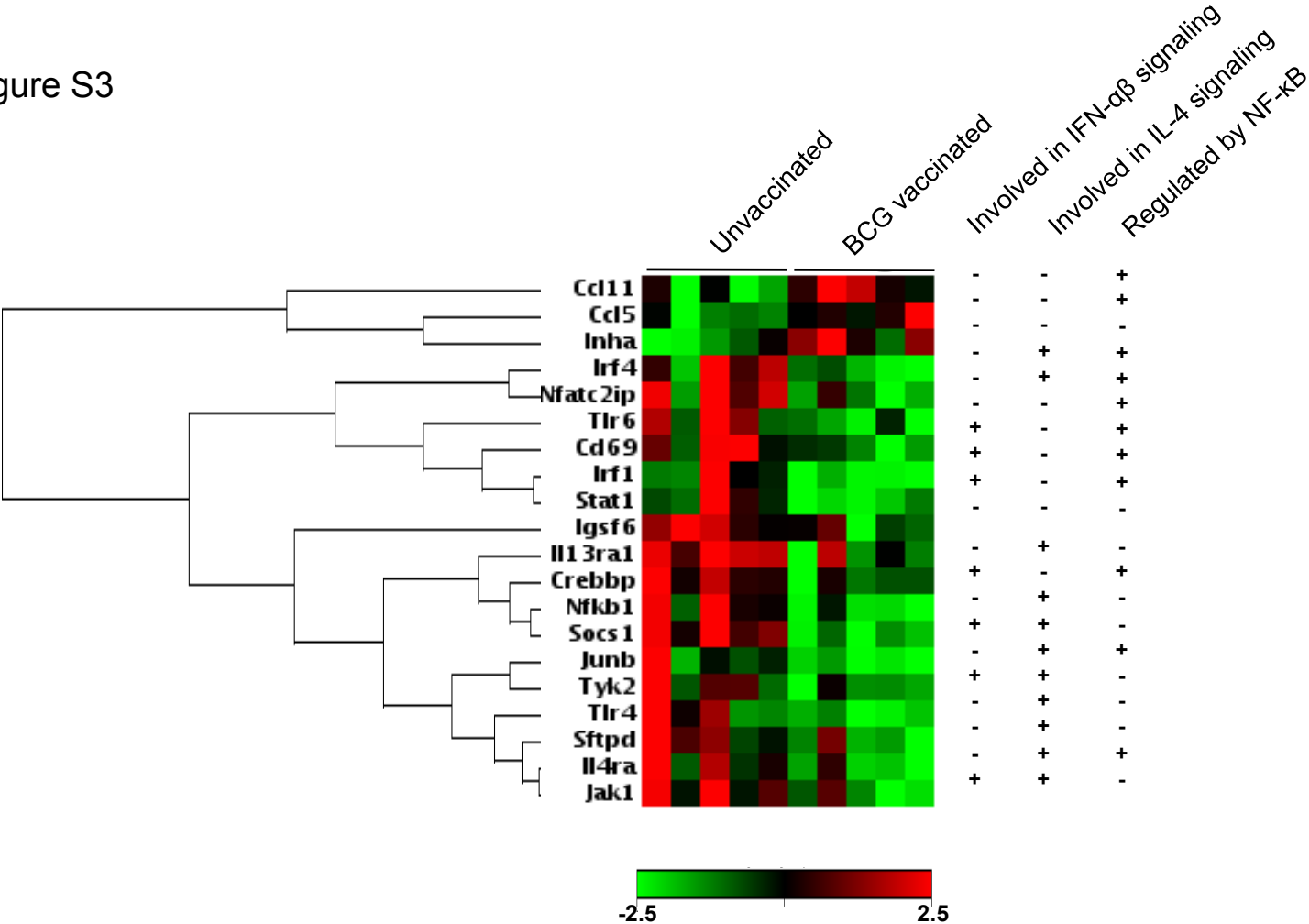

Supplement: Figure S3 — Cluster diagram of 20 genes, fold difference of which was statistically significant (P<0.05) irrespective of their fold change (look Table S1), N = 5. (PDF) [file pone.0028082.s003.pdf]
