# Supplementary material for: BCG Induces Protection against Mycobacterium tuberculosis Infection in the Wistar Rat Model
Source: PLoS One. 2011 Dec 5;6(12):e28082. doi: 10.1371/journal.pone.0028082 (PMC3230592; doi:10.1371/journal.pone.0028082)
Supplement: Table S1 — Fold regulation in the gene expression in the lung of BCG vaccinated rats compared to unvaccinated rats. Fold regulation and p value for each gene in the RT2 Profiler PCR Array Rat Th1-Th2-Th3 (Cat No. PARN-034A) has been presented. Postively and negatively regulated genes in BCG vaccinated rats compared to unvaccinated are shown in red and blue respectively. Genes for which p value was significant was taken into consideration. (PDF) [file pone.0028082.s004.pdf]

Table S1

Genes	Description	Fold regulation (BCG vaccinated / Unvaccinated)	P value (Fold difference)
Bcl3	B-cell CLL/lymphoma 3	-1.0963	0.696573
Bcl6	B-cell CLL/lymphoma 6	-1.2472	0.267927
Ccl11	Chemokine (C-C motif) ligand 11	1.7211	0.022125
Ccl5	Chemokine (C-C motif) ligand 5	1.6081	0.020024
Ccl7	Chemokine (C-C motif) ligand 7	-1.0201	0.858006
Ccr2	Chemokine (C-C motif) receptor 2	-1.5397	0.150071
Ccr3	Chemokine (C-C motif) receptor 3	-1.4346	0.149149
Ccr4	Chemokine (C-C motif) receptor 4	-1.2097	0.348117
Ccr5	Chemokine (C-C motif) receptor 5	-1.2524	0.35655
Cd28	Cd28 molecule	-1.3743	0.206961
Cd4	Cd4 molecule	-1.3201	0.299008
Cd69	Cd69 molecule	-1.3385	0.020978
Cd80	Cd80 molecule	-1.6686	0.160236
Cd86	CD86 molecule	-1.3367	0.149225
Cebpb	CCAAT/enhancer binding protein (C/EBP), beta	1.0405	0.570579
Crebbp	CREB binding protein	-1.776	0.012897
Csf2	Colony stimulating factor 2 (granulocyte-macrophage)	1.123	0.692184
Ctla4	Cytotoxic T-lymphocyte-associated protein 4	-1.4012	0.136505
Cxcr3	Chemokine (C-X-C motif) receptor 3	-1.1881	0.378557
Gata3	GATA binding protein 3	-1.6943	0.109137
Gfi1	Growth factor independent 1 transcription repressor	1.0107	0.957049
Ccr10	Chemokine (C-C motif) receptor 10	-1.0963	0.696573
Icos	Inducible T-cell co-stimulator	-1.0738	0.602329
Ifng	Interferon gamma	2.0141	0.059143
Igsf6	Immunoglobulin superfamily, member 6	-1.4149	0.034131
Il10	Interleukin 10	-1.3954	0.406909
Il12b	Interleukin 12b	-1.2576	0.337794
Il12rb2	Interleukin 12 receptor, beta 2	1.2053	0.482043
Il13	Interleukin 13	-1.0963	0.696573
Il13ra1	Interleukin 13 receptor, alpha 1	-1.3092	0.01342
Il15	Interleukin 15	-1.1116	0.356151
Il18	Interleukin 18	-1.2769	0.064647
Il18bp	Interleukin 18 binding protein	-1.1508	0.383094
Il18r1	Interleukin 18 receptor 1	-1.0473	0.596999
Il1r1	Interleukin 1 receptor, type I	-1.124	0.44618
Il2	Interleukin 2	-1.0963	0.696573
Il27ra	Interleukin 27 receptor, alpha	-1.4729	0.235637
Il2ra	Interleukin 2 receptor, alpha	-1.409	0.084662
Il4	Interleukin 4	-1.0963	0.696573
Il4ra	Interleukin 4 receptor, alpha	-1.6919	0.032173
Il5	Interleukin 5	-1.0634	0.76369
Il6	Interleukin 6	1.0234	0.837767
Il7	Interleukin 7	1.1091	0.43704
Il9	Interleukin 9	-1.0963	0.696573
Inha	Inhibin alpha	1.6464	0.016829
Irf1	Interferon regulatory factor 1	-1.9354	0.013039
Irf4	Interferon regulatory factor 4	-2.1004	0.020598
Jak1	Janus kinase 1	-1.3724	0.026247
Jak2	Janus kinase 2	-1.1429	0.109609
Jak3	Janus kinase 3	-2.2263	0.188608
Junb	Jun B proto-oncogene	-2.026	0.022494
LOC307231	Similar to nuclear factor of activated T-cells, cytoplasmic, calcineurin-dependent 1	-1.5962	0.063602
Maf	V-maf musculoaponeurotic fibrosarcoma oncogene h	-1.3877	0.077008
Mapk8	Mitogen-activated protein kinase 8	-1.1701	0.501496

Mapk9	Mitogen-activated protein kinase 9	-1.0257	0.804591
Cd27	CD27 molecule	-1.311	0.255724
Nfatc2	Nuclear factor of activated T-cells, cytoplasmic, calcineurin-dependent 2	-1.3857	0.179315
Nfatc2ip	Nuclear factor of activated T-cells, cytoplasmic, calcineurin-dependent 2 interacting protein	-1.5227	0.024226
Nfatc3	Nuclear factor of activated T-cells, cytoplasmic, calcineurin-dependent 3	-1.3238	0.056555
Nfkb1	Nuclear factor of kappa light polypeptide gene enhancer in B-cells 1	-2.083	0.008828
Ptprc	Protein tyrosine phosphatase, receptor type, C	-1.0738	0.15389
Il27	Interleukin 27	-1.3019	0.234823
Socs5	Suppressor of cytokine signaling 5	-1.0286	0.660653
Pcgf2	Polycomb group ring finger 2	-1.2805	0.294523
Sftpd	Surfactant protein D	-1.594	0.048367
Socs1	Suppressor of cytokine signaling 1	-2.5362	0.000336
Socs3	Suppressor of cytokine signaling 3	-1.7227	0.065168
Spp1	Secreted phosphoprotein 1	-2.1004	0.052306
Stat1	Signal transducer and activator of transcription 1	-1.8285	0.011303
Stat4	Signal transducer and activator of transcription 4	1.058	0.975823
Tbx21	T-box 21	-1.9408	0.067615
Dazap2	DAZ associated protein 2	1.0121	0.935981
Tgfb3	Transforming growth factor, beta 3	-1.0918	0.605199
Tlr4	Toll-like receptor 4	-1.4346	0.025019
Tlr6	Toll-like receptor 6	-1.5896	0.021118
Tmed1	Transmembrane emp24 protein transport domain containing 1	1.0334	0.813272
Tnf	Tumor necrosis factor (TNF superfamily, member 2)	1.1075	0.700099
Tnfrsf4	Tumor necrosis factor receptor superfamily, member 4	-1.0812	0.696901
Cd40	CD40 molecule, TNF receptor superfamily member 5	-1.2131	0.183233
Tnfrsf8	Tumor necrosis factor receptor superfamily, member 8	-1.2559	0.23997
Tnfsf4	Tumor necrosis factor (ligand) superfamily, member 4	-1.0963	0.696573
Cd40lg	CD40 ligand	-1.293	0.389769
Tyk2	Tyrosine kinase 2	-1.5376	0.046887
Yy1	YY1 transcription factor	-1.0767	0.446156
